# Dapagliflozin attenuates diabetes-induced diastolic dysfunction and cardiac fibrosis by regulating SGK1 signaling

**DOI:** 10.1186/s12916-022-02485-z

**Published:** 2022-09-07

**Authors:** Seul-Gee Lee, Darae Kim, Jung-Jae Lee, Hyun-Ju Lee, Ro-kyung Moon, Yong-Joon Lee, Seung-Jun Lee, Oh-Hyun Lee, Choongki Kim, Jaewon Oh, Chan Joo Lee, Yong-ho Lee, Seil Park, Ok-Hee Jeon, Donghoon Choi, Geu-Ru Hong, Jung-Sun Kim

**Affiliations:** 1 Yonsei Cardiovascular Research Institute, Yonsei University College of Medicine, Seoul, South Korea; 2 Division of Cardiology, Department of Medicine, Samsung Medical Center, Sungkyunkwan University School of Medicine, Seoul, South Korea; 3 Graduate Yonsei University, Seoul, South Korea; 4 College of Medicine, Yonsei University Seoul, Seoul, South Korea; 5 Division of Cardiology, Severance Hospital, Yonsei University College of Medicine, Seoul, South Korea; 6 Division of Cardiology, Yongin Severance Hospital, Yonsei University College of Medicine, Gyeonggi-do, South Korea; 7 Department of Cardiology, Ewha Womans University College of Medicine, Seoul Hospital, Seoul, South Korea; 8 Division of Endocrinology and Metabolism, Department of Internal Medicine, Severance Hospital, Yonsei University College of Medicine, Seoul, South Korea; 9 Cardiovascular Product Evaluation Center, Yonsei University College of Medicine, Seoul, South Korea

**Keywords:** Heart failure, Diabetes mellitus, Sodium-glucose cotransporter 2 inhibitor, Left ventricular diastolic function

## Abstract

**Background:**

Recent studies have reported improved diastolic function in patients administered sodium-glucose cotransporter 2 inhibitors (SGLT2i). We aimed to investigate the effect of dapagliflozin on left ventricular (LV) diastolic function in a diabetic animal model and to determine the molecular and cellular mechanisms underlying its function.

**Methods:**

A total of 30 male New Zealand white rabbits were randomized into control, diabetes, or diabetes+dapagliflozin groups (*n* = 10/per each group). Diabetes was induced by intravenous alloxan. Cardiac function was evaluated using echocardiography. Myocardial samples were obtained for histologic and molecular evaluation. For cellular evaluation, fibrosis-induced cardiomyoblast (H9C2) cells were obtained, and transfection was performed for mechanism analysis (serum and glucocorticoid-regulated kinase 1 (SGK1) signaling analysis).

**Results:**

The diabetes+dapagliflozin group showed attenuation of diastolic dysfunction compared with the diabetes group. Dapagliflozin inhibited myocardial fibrosis via inhibition of SGK1 and epithelial sodium channel (ENaC) protein, which was observed both in myocardial tissue and H9C2 cells. In addition, dapagliflozin showed an anti-inflammatory effect and ameliorated mitochondrial disruption. Inhibition of SGK1 expression by siRNA decreased and ENaC and Na+/H+ exchanger isoform 1 (NHE1) expression was confirmed as significantly reduced as siSGK1 in the diabetes+dapagliflozin group.

**Conclusions:**

Dapagliflozin attenuated left ventricular diastolic dysfunction and cardiac fibrosis via regulation of SGK1 signaling. Dapagliflozin also reduced macrophages and inflammatory proteins and ameliorated mitochondrial disruption.

**Supplementary Information:**

The online version contains supplementary material available at 10.1186/s12916-022-02485-z.

## Background

Sodium-glucose transporter-2 inhibitors (SGLT2is) reduce blood glucose by inhibiting glucose reabsorption in the proximal tubule and are approved for the treatment of type 2 diabetes mellitus (DM).

Recent large clinical trials reported a cardiovascular benefit of SGLT2is in type 2 DM patients [1–4]. A meta-analysis of SGLT2is in cardiovascular outcome trials showed that SGLT2is reduced the risk of heart failure (HF) hospitalization by 31%, which was consistent in patients with or without a history of HF [5]. The pathophysiological mechanism of the beneficial effect of SGLT2i is likely to be independent of glucose-lowering. Both DAPA-HF (The Study to Evaluate the Effect of Dapagliflozin on the Incidence of Worsening Heart Failure or Cardiovascular Death in Patients With Chronic Heart Failure) and the EMPEROPR Reduced trial (The Empagliflozin Outcome Trial in Patients With Chronic Heart Failure With Reduced Ejection Fraction) showed significantly reduced HF hospitalization or death rates in patients with HF, regardless of the absence of diabetes [6, 7]. In addition, emerging evidence of the cardioprotective effect of SGTL2i in doxorubicin-induced cardiomyopathy suggests a potential role of SGTL2i in the cardio-oncology field [8, 9]. Meanwhile, the mechanisms underlying the cardiovascular benefits of SGTL2i remain elusive.

Progression of diastolic dysfunction is a risk factor for HF [10]. Diastolic dysfunction predicts mortality in HF, irrespective of left ventricular (LV) ejection fraction [11, 12]. LV diastolic dysfunction results from increased myocardial stiffness and interstitial myocardial fibrosis [13]. Previous studies suggest SGLT2is may inhibit cardiac fibrosis and ameliorate diastolic dysfunction [14, 15]. However, the underlying cellular and molecular mechanisms remain to be elucidated [16, 17].

In this study, we aimed to investigate the cellular and molecular mechanisms underpinning the effect of dapagliflozin (a widely-used SGLT2i) on cardiac fibrosis and diastolic function.

## Methods

### Experimental animal model

The study protocol was approved by the local Institutional Animal Care and Use Committee (IACUC) of Yonsei University Health System (YUHS-IACUC: 2016-0157) and complies with the ARRIVE reporting guidelines. Healthy New Zealand white rabbits were purchased from Dooyeol Biotech (Dooyeol Biotech, Seoul, Korea) and were maintained under the same standard laboratory conditions, housed at room temperature with a 12-h light cycle with free access to diet and water in each cage. All animals were submitted to daily health status monitoring including weight, food intake, and general activity. All protocols followed the guidelines for the care and use of laboratory animals (National Research Council, USA). The main outcome variable was diastolic dysfunction assessed by echocardiography. Based on a previous study [18], a case number estimation had yielded a group size of *n* = 10 (8 animals + 2 reserve animals). A total of 30 male rabbits (3.0–3.5 kg, 22–24 weeks) were randomly allocated to three groups: control (*n* = 10), diabetes (*n* = 10), and diabetes + dapa (dapagliflozin, 1 mg/kg/day/P.O. for 8 weeks) (*n* = 10). Diabetic condition was induced by intravenous injection of Alloxan monohydrate (ALX, Sigma-Aldrich, St. Louis, MO, USA) at a dose of 150 mg/kg. Rabbits exhibiting a fasting blood glucose level above 200 mg/dl were diagnosed as diabetic. All rabbits were fed for a 1% cholesterol diet (Dooyeol Biotech) for 6 weeks and a normal diet for 2 weeks thereafter. After follow-up, echocardiography was performed in the prone position after anesthesia with an intramuscular injection of an appropriate mixture of Zoletil and Rompun. After echocardiography, the animals were euthanized to minimize the discomfort experienced by animals. The experimental protocol is shown in Additional file 1: Fig. S3 in detail.

### Blood chemistry

Glucose and cholesterol levels were measured in blood samples at baseline, diabetes modeling, and follow-up after 8 weeks using a Blood Glucose Monitoring System (Osang healthcare, Anyang, Korea) and DRI-CHEM 4000i (Fujifilm, Tokyo, Japan). Blood samples were drawn from the ear veins of the rabbits after they had been fasted overnight.

### Conventional echocardiography

All images were obtained using a commercial ultrasound machine (Vivid 7 Dimension; GE Vingmed Ultrasound AS, Horten, Norway) with an S10 probe (2.5 megahertz). Images were acquired from apical three-chamber, four-chamber, and two-chamber views; and short-axis views of the mitral valves, papillary muscles, and apex [19].

The left atrial end-diastolic diameter (LVEDD), left atrial end-systolic diameter (LVESD), septal, LV posterior wall thicknesses, and left atrial diameter (LAD) were measured from standard planes. The LV ejection fraction (EF) was calculated using the Teicholz formula [19]. Pulsed Doppler echocardiography of the transmitral flow was performed. The sample volume was positioned at the level of the mitral tips in the apical four-chamber view. From the transmitral recording, the peak early (E) and late diastolic filling velocities were obtained. An apical four-chamber view was also used to obtain Doppler tissue imaging of the mitral annulus. Sample volumes were placed on the septal and lateral sides of the mitral annulus. Values for systolic (S′), early (e′), and late (a′) diastolic annular velocities were obtained. Echocardiography and analysis were performed in blind conditions.

### Ultrastructure analysis using transmission electron microscopy (TEM)

The samples were cut into 1 mm squares and immediately placed in primary TEM fixation. After pretreatment, specimens were embedded with a Poly/Bed 812 kit (Polysciences, Warrington, USA) and then placed in resin and polymerized in an electron microscope oven (TD-700, DOSAKA, Kyoto, Japan) at 65 °C for 12 h. Ultrathin sections (80 nm) were placed on copper grids and double stained with 3% uranyl acetate and 3% lead citrate for 30 min and 7 min, respectively. The stained sections were then imaged using a transmission electron microscope (JEM-1011, JEOL, Tokyo, Japan) equipped with a Mega-View III CCD camera (Soft imaging system, Münster, Germany).

### Quantification of interstitial fibrosis and immunostaining

Heart tissue was fixed in 10% normal buffered formalin, embedded in paraffin, sectioned at 4μm thickness, cut on a microtome RM2235 (Leica, Wetzlar, Germany), then deparaffinized through the dewatering process. Masson’s Trichrome and Sirius Red were used to stain for collagens. Immunohistochemistry (IHC) and immunofluorescence (IF) were used to evaluate fibrosis, macrophage, or inflammation expression. Tissue sections were immunostained at 4°C overnight with antibody. IHC was used to detect α-SMA (Abcam, Cambridge, UK, ab-7817), Fibronectin (Abcam, ab-6328), TGF-β1 (Abbkine, Wuhan, China, ABP52598), 3-nitrotyrosine (Abcam, ab-61392), Receptor for advanced glycation end products (RAGE) (LifeSpan Biosciences, Seattle, USA, LS-C122375), RAM11 (DAKO, CA, USA, M0633), tumor necrosis factor-α (TNF-α) (Abcam, ab6671), NHE1 (Santa Cruz Biotechnologies, CA, USA, sc-136239), SGLT1 (Millipore, Overijse, Belgium, 07-1417), SGLT2 (Abcam, ab85626), Fis1 (Santa Cruz Biotechnologies, CA, USA, sc-376447), and Mfn1/Mitofusin1 (Santa Cruz Biotechnologies, CA, USA, sc-166644). The primary antibody was detected using a peroxidase-based kit (DAKO, Glostrup, Denmark) and visualized using DAB substrate with enhancer (DAKO). The sections were subsequently counterstained with hematoxylin (DAKO). The IHC staining was performed as previously described [20]. Digital images of the heart tissue were scanned using a SCN 400 scanner (Leica, Wetzlar, Germany), and histomorphometry was performed using LAS 4.2 software (Leica). Ten random images from 10 heart tissues per group were analyzed in a blinded procedure.

IF staining of the heart tissue to detect serum and glucocorticoid-regulated kinase 1 (SGK1) (ABCAM, ab43606) and epithelial sodium channel (ENaC) (Biorbyt, Cambridge, UK, orb100662) was performed following a published protocol [21]. The sections were washed for 10 min in 1% PBS and then incubated with FITC-conjugated secondary antibodies (Santa Cruz Biotechnologies) for 1 h in the dark at room temperature. The sections were washed in PBS for 10 min, mounted with Fluoroshield containing DAPI (ImmunoBioscience, Mukilteo, WA, USA), and stored in the dark at 4°C. Confocal microscopy was performed with an LSM 700 system (Carl Zeiss, Oberkochen, Germany).

### Cell culture and transfection

Cells of the rat cardiomyoblast cell line H9C2 were cultured in DMEM containing 10% fetal bovine serum (both from Biowest, MO, USA) supplemented with 10% non-essential amino acids, 1% 2-mercaptoethanol, and 10% penicillin (all from Gibco, Carlsbad, CA, USA). Cells were maintained at 37°C in humidified air with 5% carbon dioxide. Before treatment, the cells were washed twice with pH 7.4 phosphate-buffered saline (PBS, Gibco). The cells were incubated in 500 μM palmitate (diluted in 5% bovine serum albumin [BSA]) with or without 35mM high glucose (HG) for 24 h and then treated with 0.4 μM dapagliflozin with 10 μg/ml lipopolysaccharide (LPS) for 24 h (all from Sigma-Aldrich).

siRNA targeting rat siSGK1 (5′- AGGAGAACAUCGAGCACAATT -3′) and siControl (5′-UUCUCCGAACGUGUCACGUTT-3′) were synthesized (Bioneer, Daejeon, Korea). H9C2 cells were then transfected with the siRNAs using Lipofectamine^TM^ RNAiMAX (Invitrogen, Carlsbad, CA, USA) according to a previously described method [22].

### Reverse transcription (RT)-PCR and real-time PCR

The heart tissues and H9C2 cells of total RNA were isolated using a published procedure [23]. cDNA was synthesized using Quantitect Reverse Transcription Kit (QIAGEN, Hilden, Germany), then the cDNA was amplified using AccuPower PCR Premix (Bioneer, Daejeon, Korea) and the SYBR Green kit of the 2X Fast Q-PCR Master Mix (SMOBIO, Hsinchu City, Taiwan). Relative mRNA levels were determined by comparison with GAPDH or β-actin. The rabbit and H9C2 primers used for the target genes are shown in Additional file 1: Table S1 and S2.

### Western blot analysis

The heart tissues and H9C2 cells were lysed with RIPA buffer (Biosesang, Seongnam, Korea) containing Complete Mini and EDTA-free protease inhibitor cocktail (Roche, Basel, Switzerland). The protein samples were resolved by SDS-PAGE and then electrotransferred to an Immuno-Blot PVDF membrane (Bio-Rad, Hercules, CA, USA). Membranes were blocked with 5% skim milk (Noble Bio, Hwaseong, Korea) in 10% TBS-T for 1 h at room temperature. Membranes were incubated with primary antibodies against Fibronectin (Abcam, ab-6328), TGF-β1 (Abbkine, Wuhan, China, ABP52598), SGK1 (Abcam, ab43606), ENaC Gamma (Biorbyt, Cambridge, UK, orb100662), NHE1 (Santa Cruz Biotechnologies, CA, USA, sc-136239), TNF-α (Abcam, ab6671), IL-6 (Santa Cruz Biotechnologies), NF-kB (p65, Enzo life sciences, Farmingdale, NY, USA), and pNF-kB (p65, Santa Cruz Biotechnologies) at 4°C overnight and washed with TBS-T. They were incubated with horseradish peroxidase-conjugated secondary antibody for 1 h room temperature and then subjected to ECL (GE Healthcare, Chicago, USA) detection. GAPDH was detected on the same membrane to serve as a loading control. Densitometry analysis was performed using Image J software (National Institutes of Health, Bethesda, MD, USA).

### Statistical analysis

All data are expressed as mean ± SEM. Statistical analyses were performed using SPSS v26 (SPSS Inc., Chicago, IL, USA) and dots graphs were created using the GraphPad Prism 8.4 (GraphPad Inc., San Diego, CA, USA). When our data follow normal distribution, parametric tests otherwise nonparametric methods are used to compare the groups. *P*-values less than 0.05 were considered statistically significant.

## Results

### Metabolic parameters of animal models at baseline and 8 weeks follow-up

Baseline characteristics were comparable among the three groups; however, the fasting blood glucose level was significantly higher in the diabetes groups compared with the control group (*P* < 0.001, Table 1). At 8 weeks follow-up, body weights did not differ among the three groups, but total cholesterol, triglyceride, low-density lipoprotein, and fasting blood glucose were significantly higher in the diabetes group compared to those in the control and diabetes+dapa groups (*P* < 0.001, *P* < 0.01, *P* < 0.05, Table 1).

**Table 1:** Parameters at baseline and at the 8 weeks follow-up

	Control	Diabetes	Diabetes+dapa
**Baseline**			
Body weight, kg	3.3 ± 0.1	3.3 ± 0.1	3.4 ± 0.1
Total cholesterol (mg/dL)	29.8 ± 3.1	32.3 ± 4.1	27.9 ± 2.5
Triglyceride (mg/dL)	42.3 ± 5.9	38.2 ± 4.6	36.9 ± 5.9
High-density lipoprotein (mg/dL)	13.9 ± 2.2	14.8 ± 2.5	12.9 ± 1.9
Low-density lipoprotein (mg/dL)	7.4 ± 1.5	9.9 ± 3.0	7.6 ± 1.1
FBG (mg/dL)	133 ± 8	427 ± 36^***^	441 ± 38^***^
**At 8 week follow-up**			
Body weight, kg	3.6 ± 0.1	3.5 ± 0.2	3.8 ± 0.1
Total cholesterol (mg/dL)	268 ± 42	645 ± 34^***^	266 ± 27^†††^
Triglyceride (mg/dL)	91.4 ± 32.6	669 ± 161^***^	138 ± 35^††^
High-density lipoprotein (mg/dL)	40.6 ± 2.5	26.1 ± 2.4^***^	25.7 ± 2.9^***^
Low-density lipoprotein (mg/dL)	209 ± 36	486 ± 34^***^	213 ± 21^†††^
FBG (mg/dL)	136 ± 4	445 ± 48^***^	251 ± 36^*,†^

Values are means ± SEM (*n* = 10 per group)

*FBG* fasting blood glucose

^*^p < 0.05, ^***^p < 0.001 compared to control group, ^†^*p* < 0.05, ^††^*p* < 0.01, ^†††^*p* < 0.001 compared to diabetes group

### Attenuation of LV diastolic dysfunction in diabetes+dapa group

Baseline echocardiographic parameters were similar among the three groups. Table 2 shows comparisons of echocardiographic parameters at 8 weeks follow-up among the three groups. In the diabetes+dapa group, septal e′ and lateral e′ velocities were significantly higher than that of the diabetes group (*P* < 0.05). The E/septal e′ ratio was significantly lower in the diabetes+dapa group compared with that in the diabetes groups (*P* < 0.05).

**Table 2:** Echocardiographic parameters at 8 weeks follow-up

	Control	Diabetes	Diabetes+dapa
LAD, mm	8.8 ± 0.3	9.9 ± 0.4	9.4 ± 0.3
LVEDD, mm	14.4 ± 0.7	14.6 ± 0.7	14.3 ± 0.8
LVESD, mm	9.2 ± 0.6	9.3 ± 0.4	9.4 ± 0.3
IVSd, mm	3.6 ± 0.2	3.5 ± 0.3	3.6 ± 0.3
PWd, mm	3.2 ± 0.1	3.5 ± 0.3	3.7 ± 0.2
LVEF (%)	69 ± 2	72 ± 1	73 ± 1.6
LVFS (%)	36 ± 2	38 ± 1	39 ± 1.3
E, cm/s	61 ± 4	69 ± 10	67 ± 5.1
A, cm/s	34 ± 3	59 ± 7	45 ± 5.7
Septal e′, cm/s	9.6 ± 0.4	5.6 ± 0.7^*^	9.6 ± 0.6^†^
Lateral e′, cm/s	13.2 ± 1.0	8.2 ± 1.9^*^	13.1 ± 0.5^†^
E/septal e′	6.6 ± 0.6	12.9 ± 1.6^*^	7.2 ± 0.6^†^

Values are means ± SEM (*n* = 10 per group)

*SGLT2i* sodium-glucose cotransporter 2 inhibitor, *LAD* left atrial diameter, *LVEDD* left ventricular end-diastolic diameter, *LVESD* left ventricular end systolic diameter, *IVSd* interventricular septal end diastole thickness, *PWd* posterior wall thickness, end diastole, *LVEF* left ventricular ejection fraction, *LVFS* left ventricular fractional shortening

^*^p < 0.05 compared to control group, ^†^*p* < 0.05 compared to diabetes group

### Dapagliflozin attenuates myocardial fibrosis

**Fig. 1:**
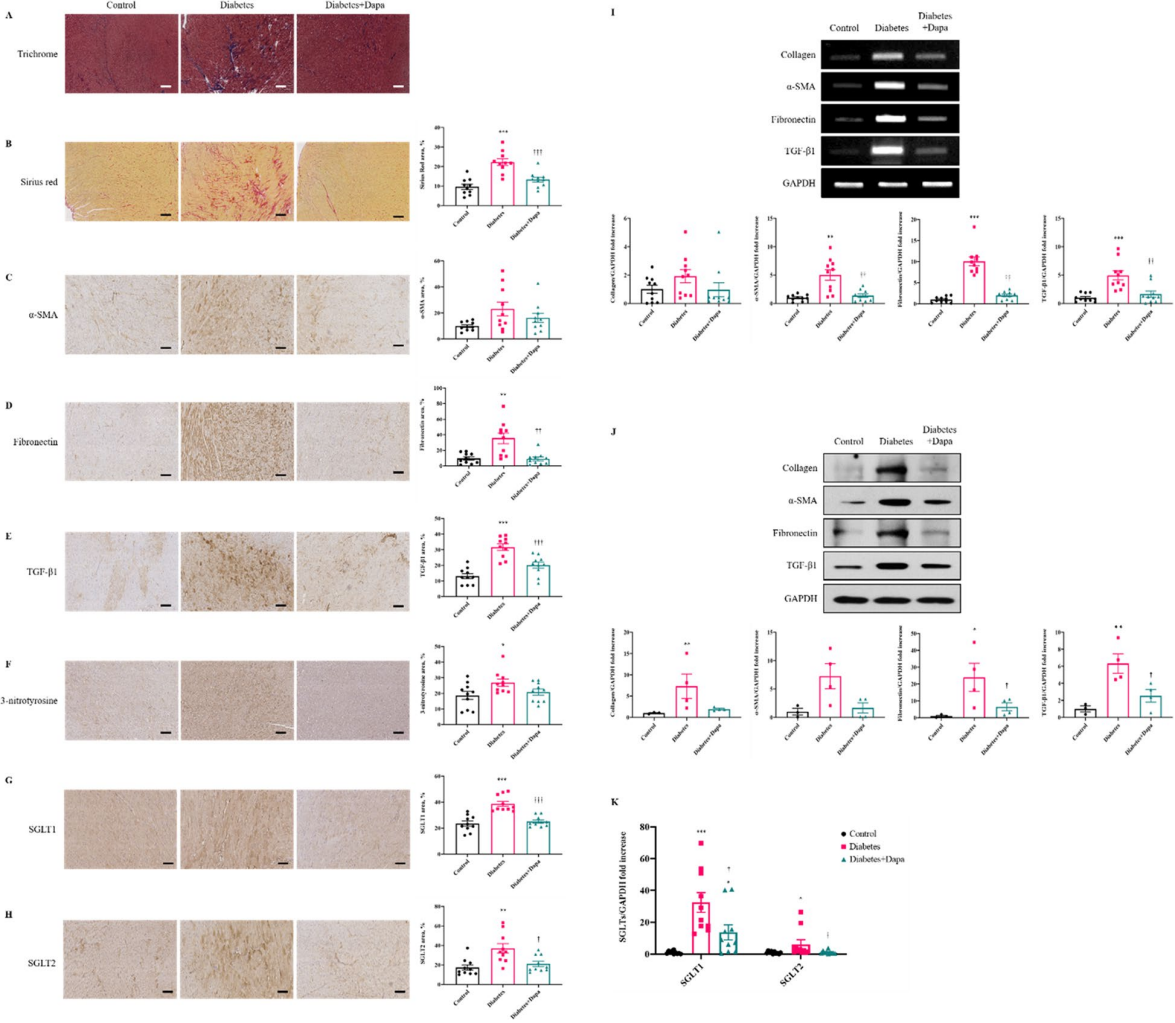
Reduction of fibrosis by dapagliflozin and morphologic changes by histological analysis. **A** Myocardial tissue was stained with Masson’s trichrome. **B** Collagen fiber deposition of the plaques is represented using Sirius Red staining. Sirius Red percentage for myocardial tissue in each group. **C**–**F** The plaques were shown using immunohistochemistry staining of α-SMA, Fibronectin, TGF-β1, and 3-nitrotyrosine. **G**, **H** The expression of SGLT1 and SGLT2 and direct effects of dapagliflozin on immunohistochemistry analysis in myocardial tissue. Scale bar = 200μm. **I** RT-PCR analyses of indicated genes in myocardial tissue. **J** Western blot analyses of indicated genes in myocardial tissue (*n* = 4 per group). **K** Real-time PCR expressions of SGLT1 and SGLT2 in myocardial tissue. Values are means ± SEM (*n* = 10 per group). ^*^p < 0.05, ^**^p < 0.01, ^***^p < 0.001 compared to control group, ^†^*p* < 0.05, ^††^*p* < 0.01, ^†††^*p* < 0.001 compared to diabetes group

A qualitative assessment by Masson’s trichrome stain revealed prominent fibrosis in the myocardium of the diabetes group (Fig. 1A). Sirius red staining revealed that the Diabetes group exhibited significantly increased myocardial fibrosis compared with the control and diabetes+dapa groups (*P* < 0.001, Fig. 1A, B), reflecting increased collagen deposition. Extracellular remodeling was assessed by immunostaining for α-SMA, fibronectin, and TGF-β1 protein levels (Fig. 1C–E). The diabetes+dapa group showed significantly decreased fibronectin and TGF-β1 compared with the diabetes group (*P* < 0.01, *P* < 0.001). Dapagliflozin significantly reduced expression of fibronectin and TGF-b1 based on RT-PCR and Western blot findings (*P* < 0.001, *P* < 0.01, *P* < 0.05, Fig. 1I, J). This finding provides visual evidence that dapagliflozin can influence the extracellular remodeling of rabbit myocardium.

To further investigate the mechanism of dapagliflozin in the attenuation of cardiac fibrosis and extracellular remodeling, we compared the expression of myocardial SGK1 protein and its downstream proteins, ENaC and NHE1.

**Fig. 2:**
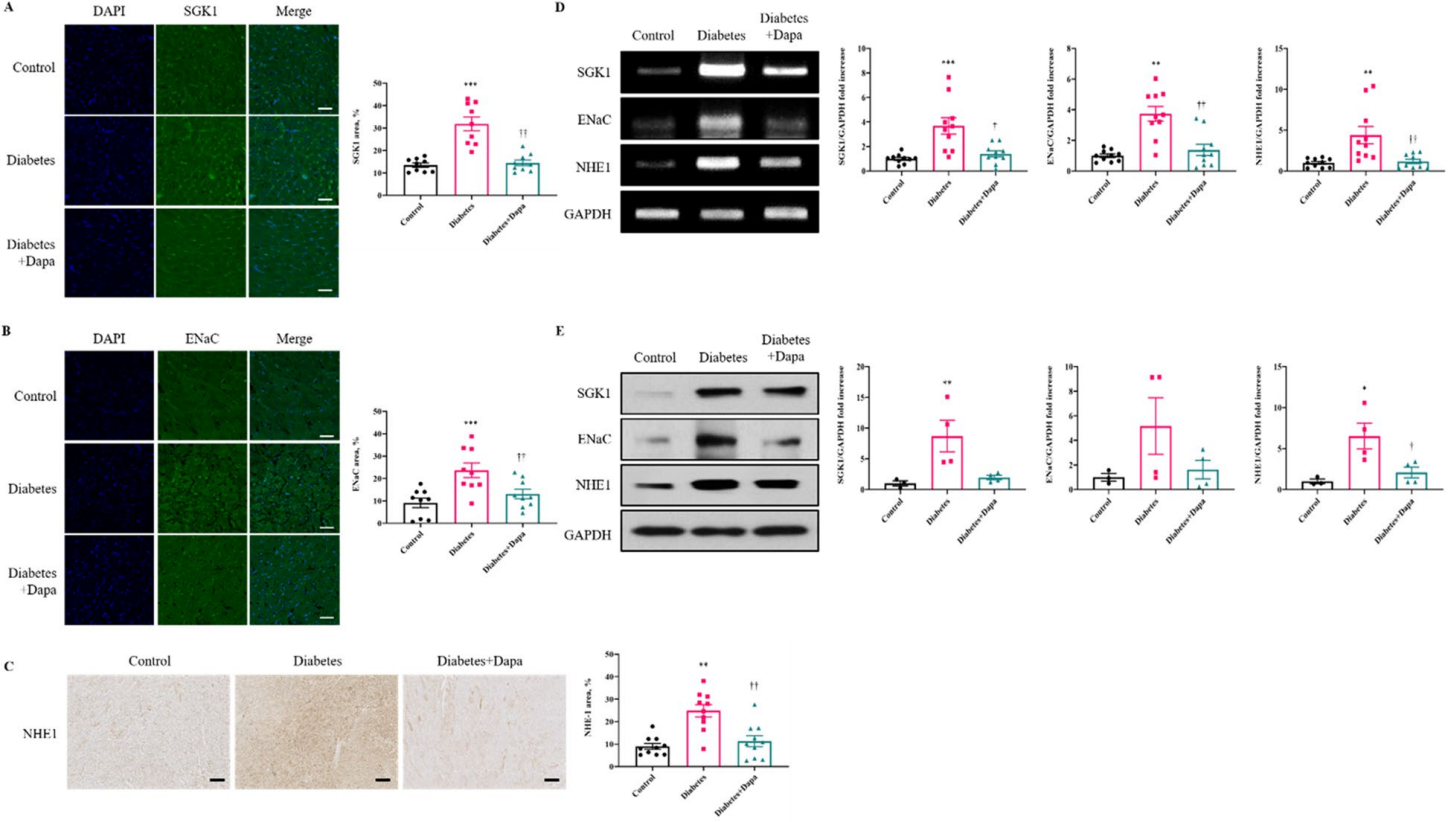
Dapagliflozin inhibits the expression of profibrotic proteins SGK1 and ENaC assessed by confocal immunofluorescence microscopy. **A** Comparison of SGK1 (green) expression in myocardial tissue. **B** Comparison of ENaC (green) expression in myocardial tissue. Relative area measurements were determined using a Zeiss LSM 700. Scale bar = 50μm. *n* = 3 each slide of 3 sections. **C** The direct effects of dapagliflozin on immunohistochemistry analysis of NHE1 in myocardial tissue (*n* = 10 per group). Scale bar = 200μm. **D** RT-PCR analyses of indicated genes in myocardial tissue (*n* = 10 per group). **E** Western blot analyses of indicated genes in myocardial tissue (*n* = 4 per group). Values are means ± SEM. ^*^p < 0.05, ^**^p < 0.01, ^***^p < 0.001 compared to control group, ^†^*p* < 0.05, ^††^*p* < 0.01 compared to diabetes group

Myocardial SGK1 and ENaC proteins were significantly decreased in the diabetes+dapa group compared with the diabetes group, and comparable to the control group, when assessed by immunofluorescence techniques (*P* < 0.001, *P* < 0.01, Fig. 2A, B). Similarly, NHE1 was significantly decreased in the Diabetes+Dapa group compared with the Diabetes group (*P* < 0.01, Fig. 2C). The RT-PCR showed a significant reduction in SGK1, ENaC, and NHE1 levels in the diabetes + dapa group compared to that in the diabetes group (*P* < 0.01, *P* < 0.05, Fig. 2D) and similarly tend to decreased expression level in Western blot (Fig. 2E).

**Fig. 3:**
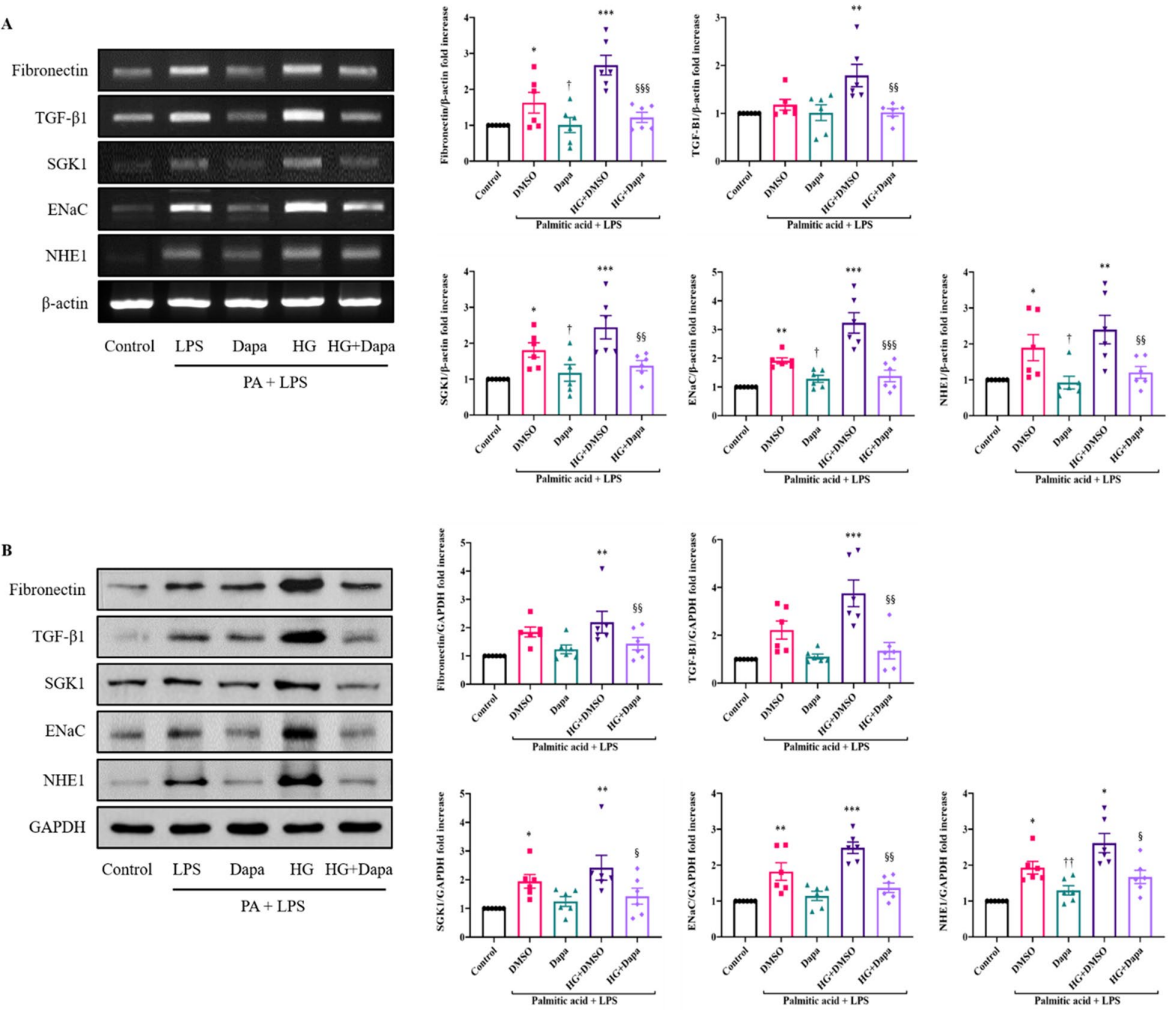
The anti-fibrotic effect of dapagliflozin in H9C2. **A** RT-PCR expression of Fibronectin, TGF-β1, SGK1, ENaC, and NHE1. **B** Western blot expression of Fibronectin, TGF-β1, SGK1, ENaC, and NHE1. Values are means ± SEM (*n* = 6). ^*^p < 0.05, ^**^p < 0.01, ^***^p < 0.001 compared to Control, ^†^*p* < 0.05, ^††^*p* < 0.01 compared to DMSO, ^§^*p* < 0.05, ^§§^*p* < 0.01, ^§§§^*p* < 0.001 compared to HG

To confirm the effect of dapagliflozin at the cellular level, hyperglycemic fibrosis was induced in cardiomyoblast H9C2 cells using RT-PCR and Western blot (Fig. 3A, B). Fibronection and TGF-β1 mRNA and protein levels were significantly lower in the H9C2 cells in the high glucose group treated with dapagliflozin than in those in the high glucose group without dapagliflozin treatment (*P* < 0.001, *P* < 0.01). SGK1, ENaC, and NHE1 mRNA and protein levels were significantly lower in the dapagliflozin-treated group compared with those in the high glucose group (*P* < 0.001, *P* < 0.01, *P* < 0.05).

**Fig. 4:**
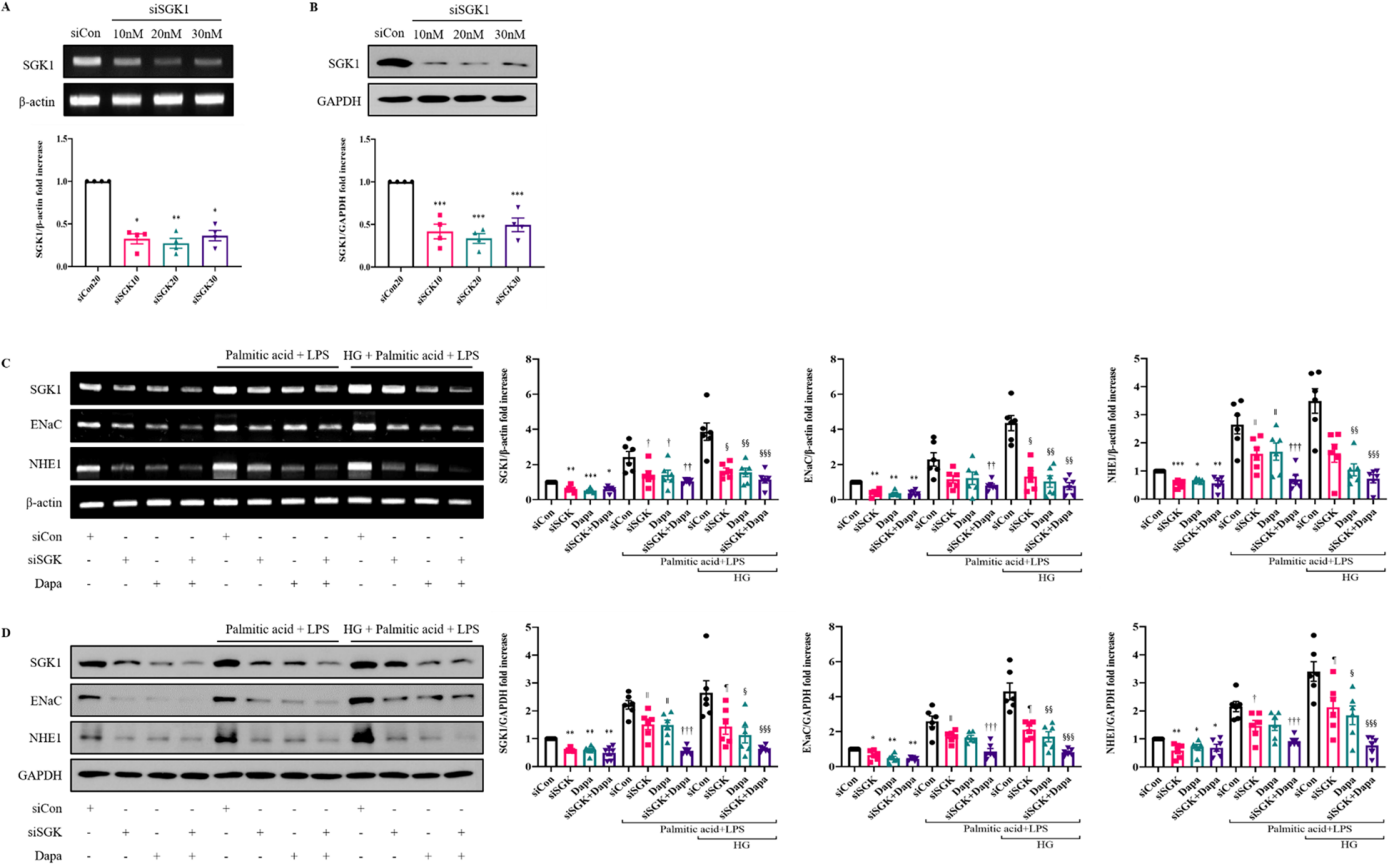
Dapagliflozin for suppression of fibrosis via SGK1, ENaC, and NHE1. **A** Representative RT-PCR bands of SGK1 in H9C2 transfected with SGK1 10-30 nM siRNA. **B** Representative Western blot bands of SGK1 in H9C2 transfected with SGK1 10-30 nM siRNA. Values are means ± SEM (*n* = 4). ^*^p < 0.05, ^**^p < 0.01, ^***^p < 0.001 compared to the siCon group. **C** RT-PCR analyses of indicated genes in H9C2. **D** Western blot analyses of indicated genes in H9C2. Values are means ± SEM (*n* = 6). ^*^p < 0.05, ^**^p < 0.01, ^***^p < 0.001 compared to the siCon group, ^†^*p* < 0.05, ^††^*p* < 0.01, ^†††^*p* < 0.001 compared to palmitic acid + LPS siCon, ^∥^p < 0.05 compared to palmitic acid + LPS siSGK + dapa, ^§^*p* < 0.05, ^§§^*p* < 0.01, ^§§§^*p* < 0.001 compared to palmitic acid + LPS HG + siCon, ^¶^*p* < 0.05 compared to palmitic acid + LPS HG + siSGK + dapa

To test whether SGK1 was directly inhibited by dapagliflozin, we assessed SGK1 expression in dapagliflozin-treated H9C2 cells. SGK1 siRNA (20 nM) effectively suppressed the transcriptional and translational level of SGK1 (*P* < 0.001, *P* < 0.01, *P* < 0.05, Fig. 4A, B). Dapagliflozin suppressed the SGK1 level and subsequently suppressed ENaC and NHE1, similar to SGK1 downregulated cells (*P* < 0.001, *P* < 0.01, *P* < 0.05, Fig 4C, D).

### Dapagliflozin reduces inflammation and ameliorates mitochondrial disruption

To explore other possible mechanisms for the cardiovascular benefit of dapagliflozin, 3-nitrotyrosine (an oxidative stress marker) expression was compared between the three groups. 3-nitrotyrosine expression was significantly lower in the diabetes+dapa group than in the Diabetes group, and comparable to the control group (*P* < 0.05, Fig. 1F). In the expression of macrophage and inflammation protein markers, including RAM11, RAGE, and TNF-α, using immunohistochemistry analysis. RAM11, RAGE, and TNF-α expression were significantly lower in the Diabetes+Dapa group compared with the Diabetes group, and comparable to the Control group (Additional file 1: Fig. S1 A-C). Decreased expression of inflammatory markers, TNF-α, IL-6, and NF-kB, was observed in dapagliflozin-treated H9C2 cells compared with the high glucose group. (Additional file 1: Fig. S2 A-B).

**Fig. 5:**
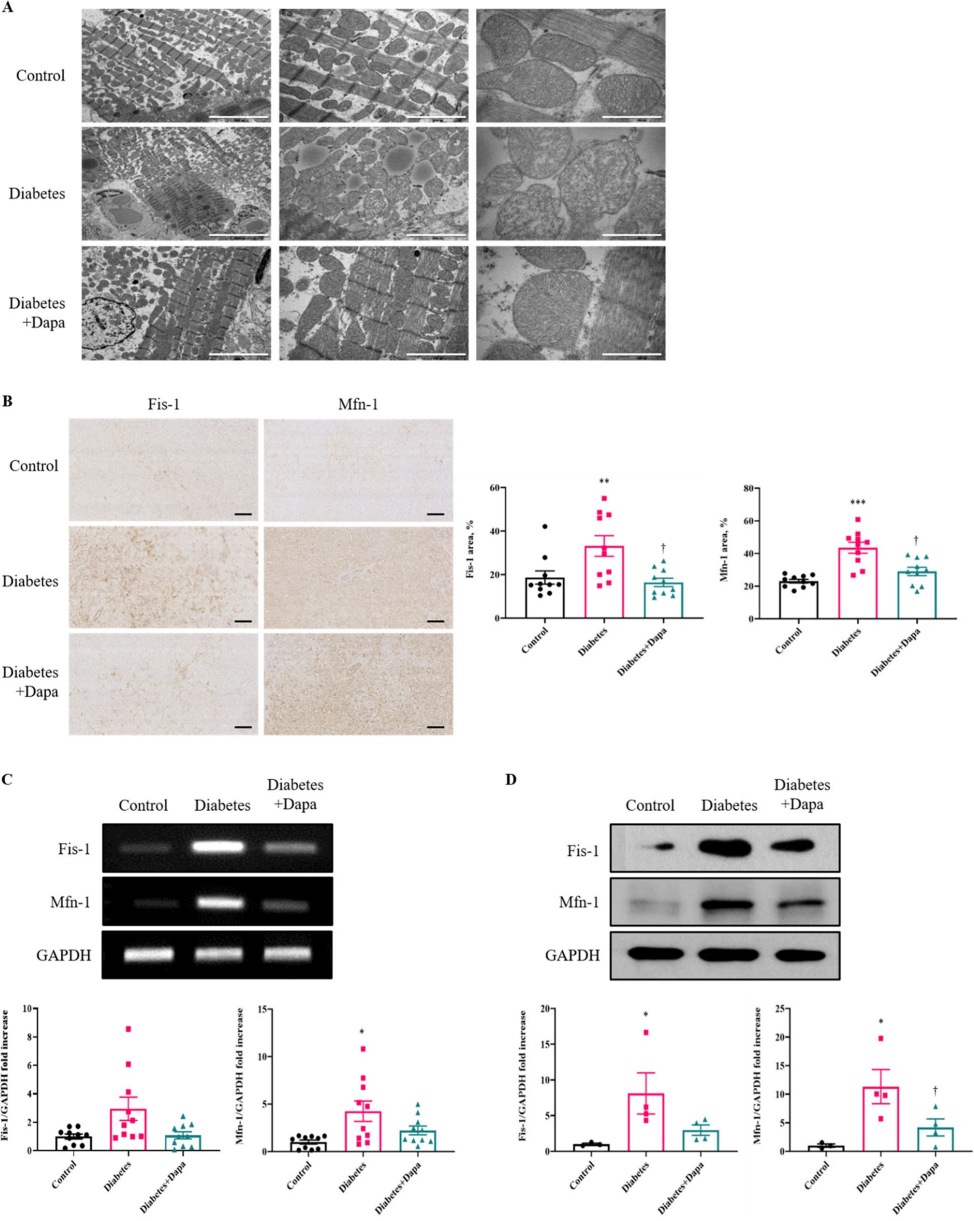
Protective effect of dapagliflozin for mitochondrial cristae in myocardial tissue. Comparison of mitochondrion changes. **A** Transmission electron microscopy images of myocardial tissue treated with dapagliflozin. Scale bar = 5μm, 2μm, and 500nm from low to high magnification. **B** Immunohistochemistry analysis of Fis-1 and Mfn-1 expression and quantification of myocardial tissue cross-sectional area. Scale bar = 200μm. **C** RT-PCR analyses of indicated genes in myocardial tissue. **D** Western blot analyses of indicated genes in myocardial tissue (*n* = 4 per group). Values are means ± SEM (*n* = 10 per group). ^*^p < 0.05, ^**^p < 0.01, ^***^p < 0.001 compared to the Control group, ^†^*p* < 0.05 compared to the diabetes group

To address possible mechanisms for decreased 3-nitrotyrosine expression in the diabetes+dapa group, mitochondria structures were analyzed. In the diabetes group, mitochondria showed a disrupted cristae structure with diminution of matrix electron density, loss and fusion of cristae, and mitochondrial fragmentation compared with the control group (Fig. 5A). In the diabetes+dapa group, the cristae structure in the mitochondria was preserved and a lesser degree of the diabetes-induced ultrastructural anomalies of the mitochondria was noted. Additionally, to further investigate the impact of dapagliflozin on mitochondrial dysfunction, we analyzed the expression levels of mitofission or mitofusion proteins, Fis1 and Mfn1. Levels of Fis-1 land Mfn-1 were significantly reduced in the Diabetes+Dapa group compared with the diabetes group (*P* < 0.05, Fig. 5B). RT-PCR and Western blot analysis also showed similar results (*P* < 0.05, Fig. 5C, D).

In the expression levels of SGLT1 and SGLT2 in heart tissue, both SGLT1 and SGLT2 were significantly higher expressions in the diabetes group when compared to those in the control group (*P* < 0.001, *P* < 0.01, *P* < 0.05, Fig. 1G–H and K).

## Discussion

In the present study, we demonstrated attenuation of LV diastolic dysfunction using a diabetic rabbit model and evaluated the underlying mechanisms. Our results showed that dapagliflozin (1) attenuated myocardial fibrosis through SGK1/ENaC/NHE1 signaling and (2) reduced myocardial inflammation, and ameliorated mitochondrial disruption.

The most important finding of our study is that we identified the potential mechanisms underlying the cardiovascular benefit of dapagliflozin. In our study, dapagliflozin attenuated LV diastolic dysfunction in a diabetic rabbit model. This finding is consistent with previous clinical and animal studies, which reported improved diastolic function with SGLT2i [16, 17, 24, 25]. The dapagliflozin-treated group showed attenuation of myocardial fibrosis with reduction of SGK1/ENaC/NHE1 proteins in myocardial tissue and H2C2 cells. SGK1 is an emerging mediator of cardiac fibrosis, which activates the ENaC proteins responsible for promoting fibrosis and upregulating NHE1 activity, which are key factors in cardiac remodeling [18, 26]. A previous study using a murine model reported activation of SGK1-induced adverse ventricular remodeling, fibrosis, and increased size of cardiomyocytes, suggesting that SGK1 is a key mediator of cardiac remodeling [27–29].

In addition to inhibition of fibrosis, our results also demonstrate that dapagliflozin reduces inflammation and ameliorates mitochondrial disruption attenuation. This finding could contribute to the attenuation of LV diastolic dysfunction. Decreased mitochondrial function, increased reactive oxygen species production, and inflammation are hallmarks of a diabetic heart [30]. A previous report demonstrated that SGTL2i prevents mitochondrial dysfunction, reflected by reduced H_2_O_2_ release and increased ATP synthesis, which supports our finding on mitochondria structure [31].

The anti-inflammatory effect of SGLT2 inhibitors, including dapagliflozin, has been reported by reducing the expression of the NLRP3 inflammasome, IL-1β, IL-6, and TNF-α [3, 32–34]. In an angiotensin II stressed diabetic mouse model, dapagliflozin decreased intracellular calcium transients, thereby reducing production of reactive oxygen species and inflammation [35]. Similarly, a recent study reported attenuation of diastolic dysfunction in a diabetic model with nutraceuticals like quercetin and boswellic acid through NLRP3 inflammasome and cytokines, which showed a potential effect against cancer cell survival and chemoresistance [36].

The possible anti-inflammatory mechanism of dapagliflozin is activation of adenosine mono-phosphate kinase (AMPK) with downstream inhibition of the Na+ /H+ Exchanger-1 (NHE-1) for the attenuation of NLRP3 inflammasome activation [37]. Furthermore, a recent study suggested that dapagliflozin may activate mTORC2, leading to the activation of Akt and FOXO3 [36] and depends on AMPK and mTOR activation. Similar to previous studies, our results showed an anti-inflammatory effect of dapagliflozin in diabetic cardiomyopathy. Inflammatory markers, RAM11, RAGE, and TNF-α were significantly reduced in the Diabetes+Dapa group compared to those in the Diabetes group (Additional file 1: Fig. S1-S2).

This study is meaningful in demonstrating the favorable effect of dapagliflozin and elucidates the mechanism underlying SGLT2 inhibition in diastolic heart failure using a diabetic rabbit model. Dapagliflozin is a known selective SGTL2 inhibitor, but also has a relatively weak inhibitory activity for SGLT1 [38, 39]. We tested the expression of SGLT1 and SGLT2 by immunostaining rabbit myocardial tissue (Fig. 1G, H). In the Diabetes+Dapa group, expression of SGLT1 and SGLT2 were significantly reduced compared with the diabetes group. This finding is consistent with the inhibitory mechanism of dapagliflozin in an in-vivo model.

**Fig. 6:**
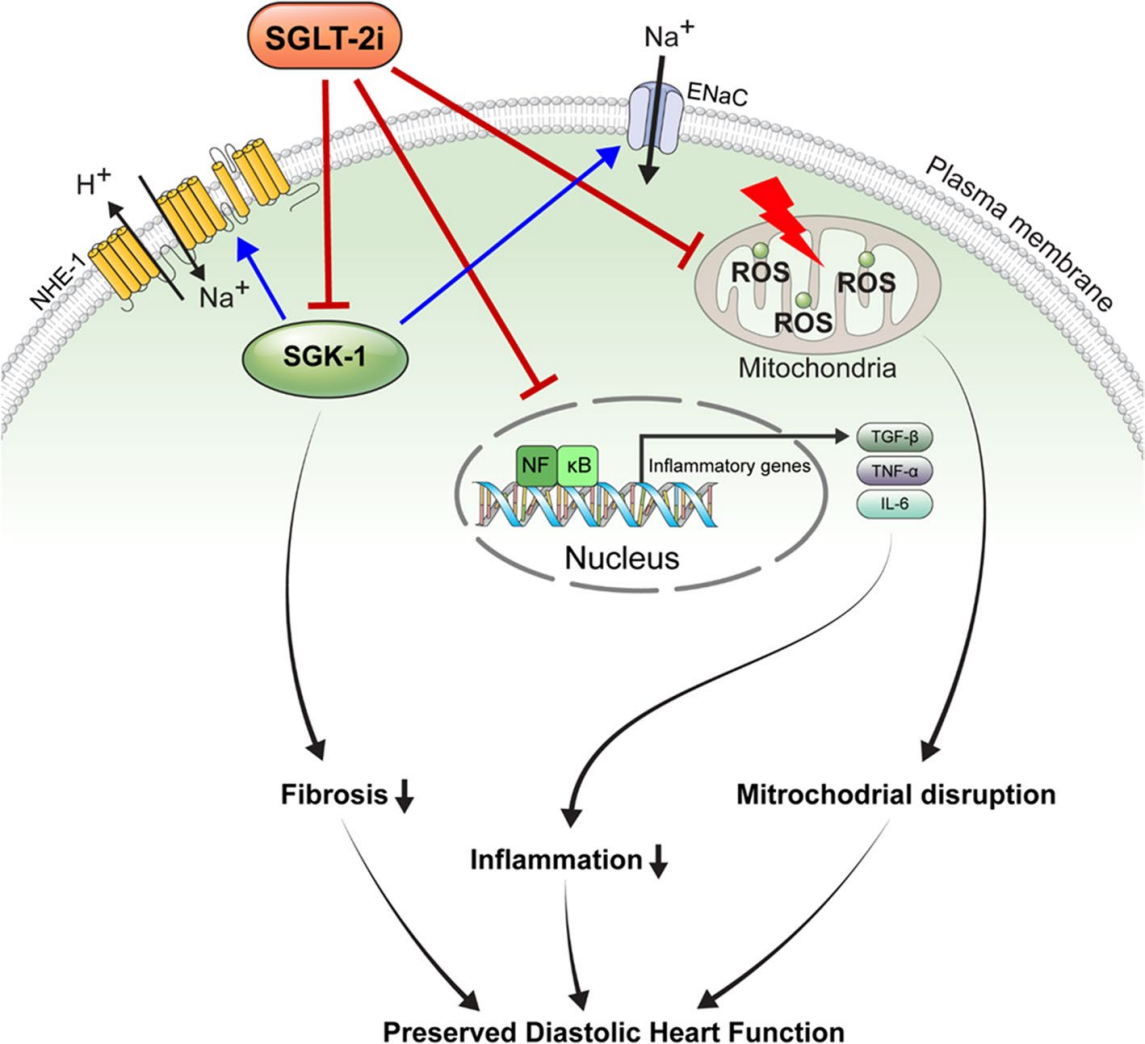
Summary of pathways contributing to suppressed cardiac fiberization in a diabetes rabbit model

There are several limitations to our study. First, our study lacks data on the effect of dapagliflozin on myocardial metabolism and mitochondrial function. Second, the present study did not include the long-term effect of dapagliflozin. Third, we assessed the effect of dapagliflozin on a diabetic model; whether dapagliflozin exerts a cardioprotective effect via the same mechanisms in a non-diabetic model needs to be explored. Despite these limitations, our study is valuable in demonstrating that dapagliflozin attenuates cardiac fibrosis via SGK1 signaling and ameliorates diastolic dysfunction in a diabetic rabbit model. Our results convey important therapeutic benefits beyond lowering blood glucose and simple intravascular volume loss by increasing the urinary excretion of glucose and sodium (Fig. 6).

## Conclusion

In conclusion, dapagliflozin attenuated left ventricular diastolic dysfunction and cardiac fibrosis via regulation of SGK1 signaling in a rabbit diabetic model. In addition, dapagliflozin reduced inflammation and ameliorated mitochondrial disruption in the myocardium. Our study may contribute to broadening the scopes of understanding the mechanism of cardiovascular benefit of SGLT2 inhibitor.

## Supplementary Information

**Additional file 1: Figure S1.** Anti-inflammatory effects of dapagliflozin on immunohistochemistry analysis. A-C The expression of RAM11, RAGE and TNF-α in the myocardium was detected by immunostaining. Values are means ± SEM (*n* = 10 per group). Scale bar = 200 μm. ^***^p < 0.001 compared to Control group, ^††^*p* < 0.01, ^†††^*p* < 0.001 compared to Diabetes group. **Figure S2.** Dapagliflozin significantly decreased inflammation markers in H9C2. A RT-PCR expression of TNF-α and IL-6. Comparisons of relative mRNA expression, normalized to expression of β-actin. B Western blot expression of pNF-kB, NF-kB, TNF-α and IL-6. Representative data showing protein expression, normalized to expression of GAPDH. Values are means ± SEM (*n* = 6). ^*^p < 0.05, ^**^p < 0.01, ^***^p < 0.001 compared to Control, ^†^*p* < 0.05 compared to DMSO, ^§^*p* < 0.05, ^§§^*p* < 0.01 compared to HG. GAPDH, glyceraldehyde 3-phosphate dehydrogenase; IL-6, interlukin-6; NF-κB, Nuclear factor kappa-light-chain-enhancer of activated B cells; TNF-α, tumor necrosis factor-α. **Figure S3.** Schematic design of the study protocol. Animals were randomized to Control, Diabetes, Diabetes+Dapa groups. Diabetic condition was induced for animals in Diabetes and Diabetes+Dapa groups. Dapagliflozin (1mg/kg/day) treated depending on their group assignment, for 8 weeks. After follow up echocardiography, animals were subsequently sacrificed and tissue samples were collected for assessment of histological and molecular remodeling. **Table S1.** List of rabbit primers. **Table S2.** List of H9C2 primers.

## Data Availability

The datasets used and/or analyzed during the current study are available from the corresponding author on reasonable request.

## Abbreviations

ALX: Alloxan monohydrate
BSA: Bovine serum albumin
DM: Diabetes mellitus
EF: Ejection fraction
ENaC: Epithelial sodium channel
HF: Heart failure
HG: High glucose
IF: Immunofluorescence
IHC: Immunohistochemistry
LAD: Left atrial diameter
LPS: Lipopolysaccharide
LV: Left ventricular
LVEDD: Left atrial end-diastolic diameter
LVESD: Left atrial end-systolic diameter
NHE1: Na+/H+ exchanger isoform 1
RAGE: Receptor for advanced glycation end products
RT: Reverse transcription
SGK1: Serum and glucocorticoid-regulated kinase 1
SGLT2i: Sodium-glucose cotransporter 2 inhibitor
TEM: Transmission electron microscopy
TNF-α: Tumor necrosis factor-α
