# Supplementary material for: Dapagliflozin attenuates diabetes-induced diastolic dysfunction and cardiac fibrosis by regulating SGK1 signaling
Source: BMC Med. 2022 Sep 7;20:309. doi: 10.1186/s12916-022-02485-z (PMC9450279; doi:10.1186/s12916-022-02485-z)
Supplement: Supplementary file 1 — Additional file 1: Figure S1. Anti-inflammatory effects of dapagliflozin on immunohistochemistry analysis. A-C The expression of RAM11, RAGE and TNF-α in the myocardium was detected by immunostaining. Values are means ± SEM (n = 10 per group). Scale bar = 200 μm. ***p < 0.001 compared to Control group, ††p < 0.01, †††p < 0.001 compared to Diabetes group. Figure S2. Dapagliflozin significantly decreased inflammation markers in H9C2. A RT-PCR expression of TNF-α and IL-6. Comparisons of relative mRNA expression, normalized to expression of β-actin. B Western blot expression of pNF-kB, NF-kB, TNF-α and IL-6. Representative data showing protein expression, normalized to expression of GAPDH. Values are means ± SEM (n = 6). *p < 0.05, **p < 0.01, ***p < 0.001 compared to Control, †p < 0.05 compared to DMSO, §p < 0.05, §§p < 0.01 compared to HG. GAPDH, glyceraldehyde 3-phosphate dehydrogenase; IL-6, interlukin-6; NF-κB, Nuclear factor kappa-light-chain-enhancer of activated B cells; TNF-α, tumor necrosis factor-α. Figure S3. Schematic design of the study protocol. Animals were randomized to Control, Diabetes, Diabetes+Dapa groups. Diabetic condition was induced for animals in Diabetes and Diabetes+Dapa groups. Dapagliflozin (1mg/kg/day) treated depending on their group assignment, for 8 weeks. After follow up echocardiography, animals were subsequently sacrificed and tissue samples were collected for assessment of histological and molecular remodeling. Table S1. List of rabbit primers. Table S2. List of H9C2 primers. [file 12916_2022_2485_MOESM1_ESM.docx]

**Additional files**

**ADDITIONAL FIGURE LEGENDS**

**Figure S1. Anti-inflammatory effects of dapagliflozin on immunohistochemistry analysis.**

**A-C** The expression of RAM11, RAGE and TNF-α in the myocardium was detected by immunostaining. Values are means ± SEM (n = 10 per group). Scale bar = 200 μm. ^***^*p* < 0.001 compared to Control group, ^††^*p* < 0.01, ^†††^*p* < 0.001 compared to Diabetes group.

**Figure S2. Dapagliflozin significantly decreased inflammation markers in H9C2.**

**A** RT-PCR expression of TNF-α and IL-6. Comparisons of relative mRNA expression, normalized to expression of β-actin. **B** Western blot expression of pNF-kB, NF-kB, TNF-α and IL-6. Representative data showing protein expression, normalized to expression of GAPDH. Values are means ± SEM (n = 6). ^*^*p* < 0.05, ^**^*p* < 0.01, ^***^*p* < 0.001 compared to Control, ^†^*p* < 0.05, ^††^*p* < 0.01 compared to DMSO, ^§^*p* < 0.05, ^§^*p* < 0.01 compared to HG. GAPDH, glyceraldehyde 3-phosphate dehydrogenase; IL-6, interlukin-6; NF-κB, Nuclear factor kappa-light-chain-enhancer of activated B cells; TNF-α, tumor necrosis factor-α.

**Figure S3. Schematic design of the study protocol.**

Animals were randomized to Control, Diabetes, Diabetes+Dapa groups. Diabetic condition was induced for animals in Diabetes and Diabetes+Dapa groups. Dapagliflozin (1mg/kg/day) treated depending on their group assignment, for 8 weeks. After follow up echocardiography, animals were subsequently sacrificed and tissue samples were collected for assessment of histological and molecular remodeling.

**Table S1. List of rabbit primers.**

| **Gene** | **Forward primer** | **Reverse primer** |
| --- | --- | --- |
| GAPDH | AGGTCATCCACGACCACTTC | GTGAGTTTCCCGTTCAGCTC |
| α-SMA | TGCTGTCCCTCTATGCCTCT | GAAGGAATAGCCACGCTCAG |
| Collagen | ATCAGGGACTTCCTGGTCCT | GTCCTTTCTGGCCATCATGT |
| Fibronectin | TTGCAACCCACAGTGGAATA | TTCATTGGTCCGGTCTTCTC |
| TGF-β1 | TGCTTCAGCTCCACAGAGAA | CTTGCTGTACTGGGTGTCCA |
| SGK1 | GAACCACGGGCTCGTTTCTAT | GCAGGCCATACAGCATCTCAT |
| ENaC | ACGAGACCATCCTCAGCACT | TCCTGCCGATGGATTAGAAC |
| NHE1 | CTGTCAGGGTGTGAGCAGAA | TGCGCTTTTCTCTTCCGTAT |
| Fis-1 | CGGAGCAAGTACAACGATGA | GCCCTGTCGTACTCTTGAGC |
| Mfn-1 | CCCTTCTGTTTTGGCAGGTA | AGGAAGCGTAACTGGAGCAA |
| Real-time GAPDH | TGACGACATCAAGAAGGTGGTG | GAAGGTGGAGGAGTGGGTGTC |
| Real-time SGLT1 | TCCTCACCCTTTGGTACTGG | ACAGGATACGGCTCACCATC |
| Real-time SGLT2 | GCCTTATTCCCGAGTTCTCC | GAGGCCAGAGCAGAAGAAGA |

**Table S2. List of H9C2 primers.**

| **Gene** | **Forward primer** | **Reverse primer** |
| --- | --- | --- |
| β-actin | GTGGGCGCTCTAGGCACAA | CTCTTTGATGTCACGCACGATTTC |
| SGK1 | TGCTCTATGGCCTGCCTCCGTTCT | GTCACTGGGCCCGCTCACATTTG |
| NHE1 | CACGCTGTGGAATGAT | GAAGATGTCCGAGATGC |
| Fibronectin | TCAGCTGTACCATTGCAAATC | TGGTGTCCTGATCATTGCAT |
| TNF-α | AGATGTGGAACTGGCAGAGG | CCCATTTGGGAACTTCTCCT |
| IL-6 | TTGACAGCCACTGCCTTCCC | CGGAACTCCAGAAGACCAGAGC |
| TGF-b1 | GCAACAACGCAATCTATGAC | CCTGTATTCCGTCTCCTT |
| SGLT1 | GACTGATTCTCGGCTTCCTG | GTGAGGAGGGAGATGACCAA |
| SGLT2 | TTCTGTCATCGCACTCTTGG | GATCCTTGGACACCGTCAGT |
| ENaC | CGTCAGTGGCACAAAGCCAA | GAGAGCCTCCTCAAACCATG |

**Figure S1**

**
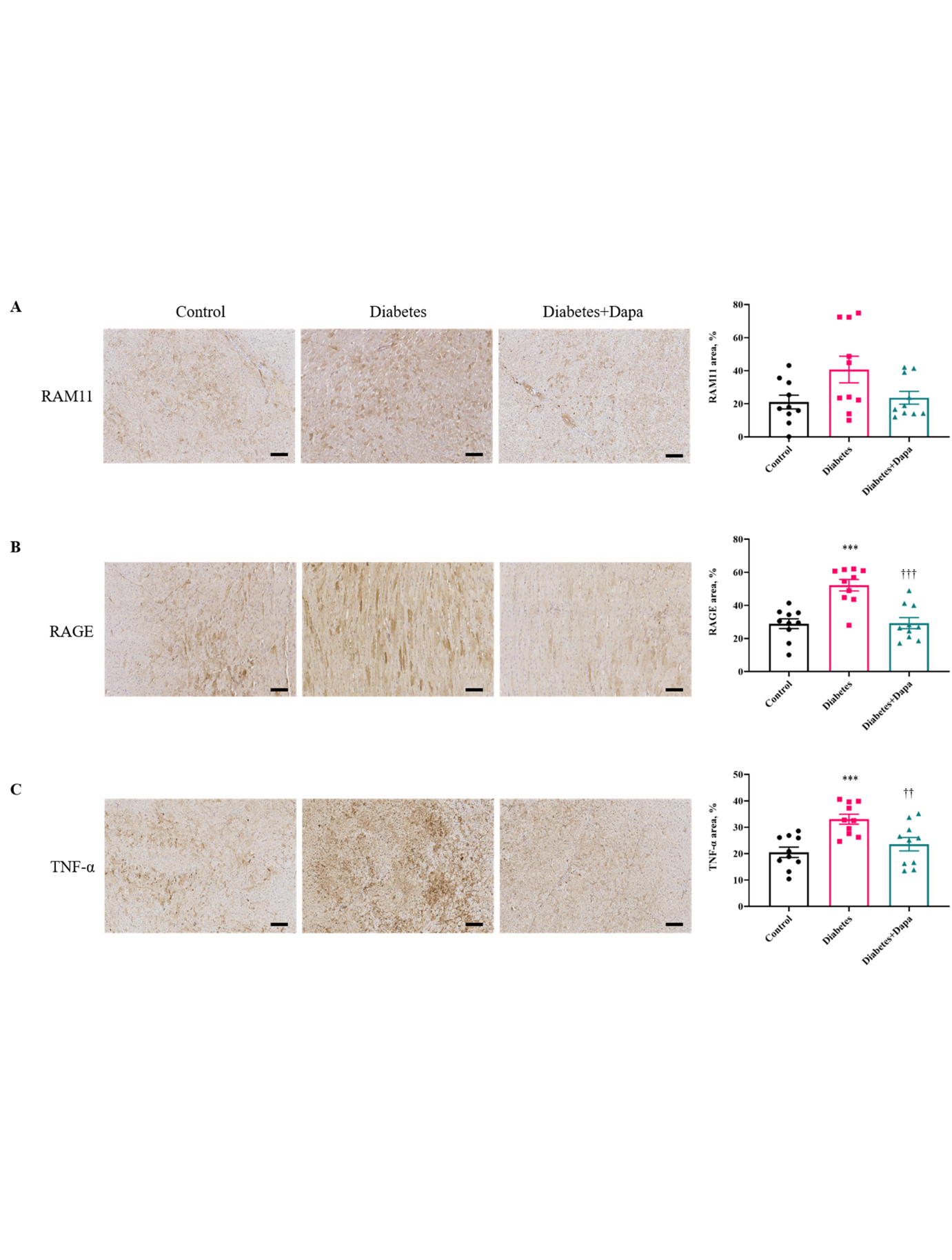
**

**Figure S2**

**
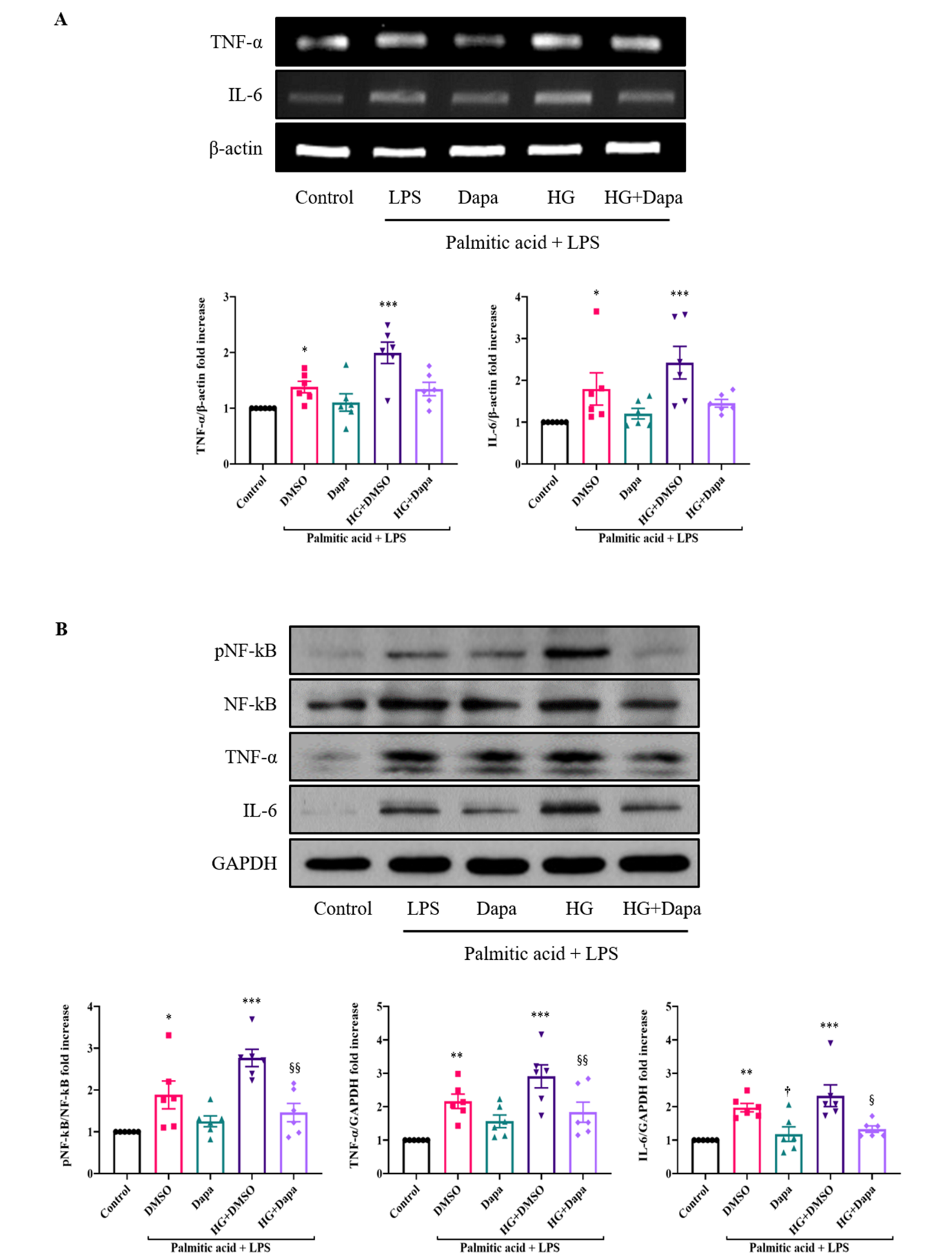
**

**Figure S3**

**
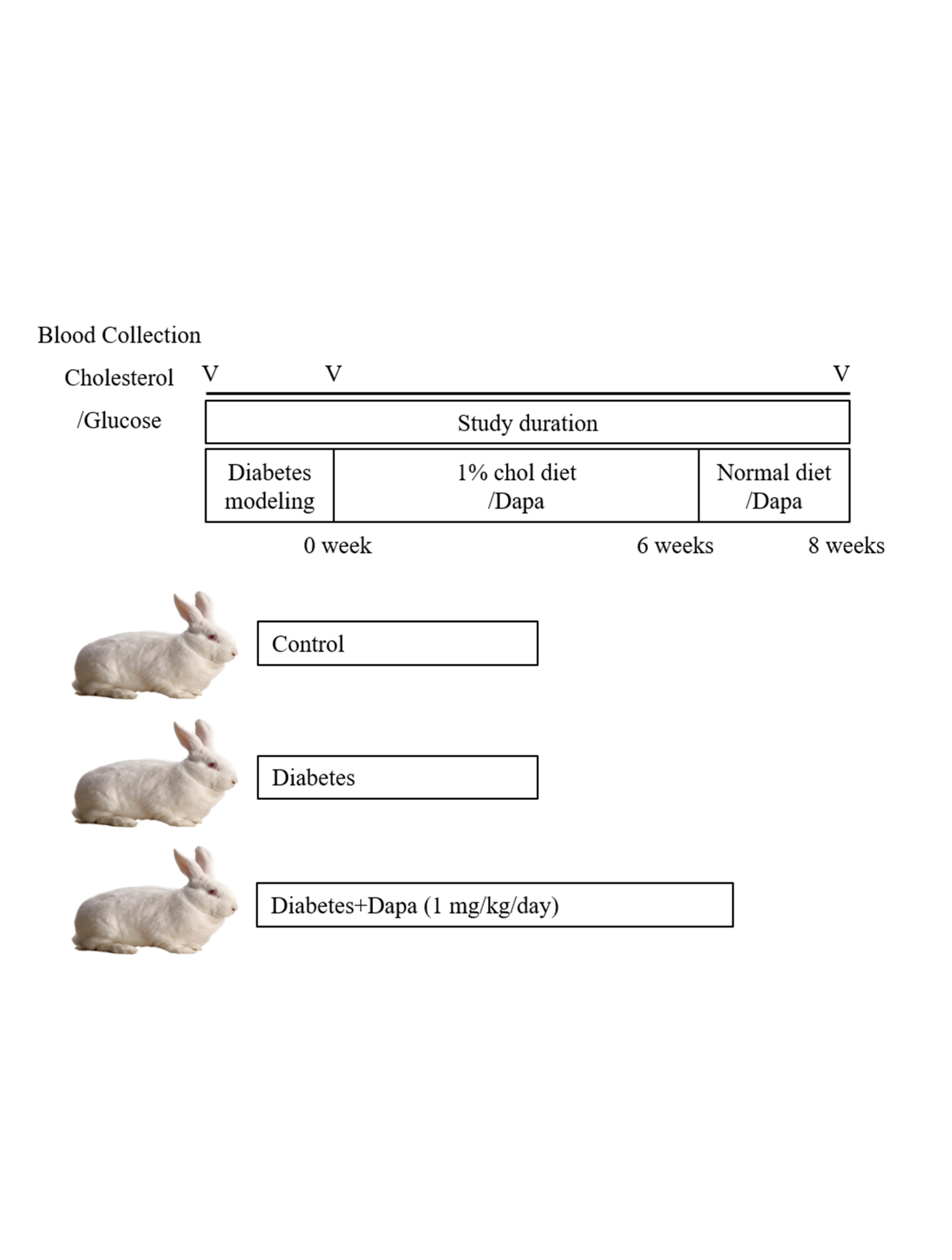
**
